# Indentation-induced plastic behaviour of nanotwinned Cu/high entropy alloy FeCoCrNi nanolaminate: an atomic simulation

**DOI:** 10.1039/d0ra00518e

**Published:** 2020-03-02

**Authors:** Hui Feng, Jingwen Tang, Haotian Chen, Yuanyuan Tian, Qihong Fang, Jia Li, Feng Liu

**Affiliations:** State Key Laboratory of Advanced Design and Manufacturing for Vehicle Body, Hunan University Changsha 410082 PR China fangqh1327@hnu.edu.cn lijia123@hnu.edu.cn; State Key Laboratory of Powder Metallurgy, Central South University Changsha 410083 PR China fengliu@csu.edu.cn

## Abstract

Using large-scale molecular dynamics (MD) simulations, the effects of interface and layer number in the nanoindentation response of experimentally observed nanotwinned Cu/high entropy alloy (HEA) FeCoCrNi nanolaminate are studied. The dislocations are nucleated and emitted, which are more limited to the first twinning layer > second twinning layer > HEA layer. The stacking fault strengthening is pronounced due to the obvious difference of stacking fault energy between Cu and HEA, which can be rarely observed from the previous work in traditional alloys and metals. After the indentation induced deformation, the nanotwinned Cu/HEA FeCoCrNi nanolaminates for different layer numbers generate a mass of Shockley partial dislocations to cause the good plasticity, attributed to the strong strain gradient effect. The strong layer number and interface structure effects found here can provide insight for the design of advanced nanolaminate with high strength and good plasticity.

## Abstract 2

Using large-scale molecular dynamics (MD) simulations, the effects of interface and layer number in the nanoindentation response of experimentally observed nanotwinned Cu/high entropy alloy (HEA) FeCoCrNi nanolaminate are studied.

## Graphical abstract

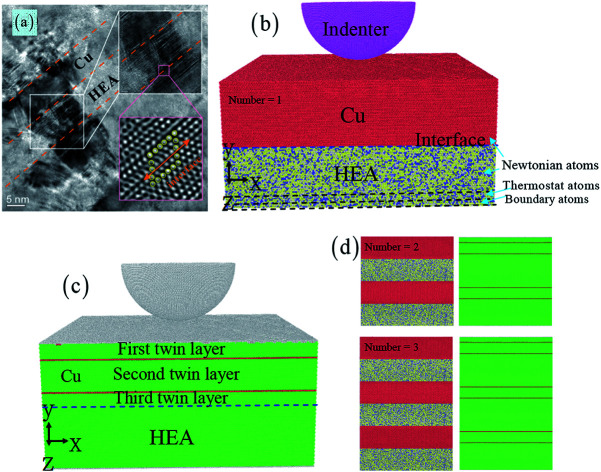

## 1. Introduction

The nanolaminated metals have received wide attention due to their outstanding mechanical properties,^1–5^ which cannot be attained with today's materials. Frequently, combining two or more dissimilar metals forms nanomaterials with an unusually high density of bimetal interfaces,^5–7^ to meet the extreme environments, such as high temperatures, radiation, stresses and large strains, above and beyond that possible with their constituents alone.

A large number of experimental studies at the nanoscale have focused on the interface structures and deformation mechanisms as well as mechanical properties.^8–10^ For example, the failure response of bulk Cu/Nb nanocomposites under planar shock loading contradicts the general thinking of failure starting from interfaces, indicating the stable Cu/Nb interfaces under dynamic loading.^11^ The deformation mechanism by kink band formation in bulk Cu/Nb nanolaminates depends strongly upon length scale and allows for large compressive strain and high stress without leading to crack formation.^12^ The strengthening capability in a micro-pillar of graphene/Al nanolaminated composites under uniaxial compression can be enhanced by either orienting the layers parallel with the loading direction or raising the concentration.^13^ Subsequently, the remarkable strain hardening and high strength of the bulk graphene/Cu nanolaminated composites are reported, due to that the dislocation storage ability can be considerably improved through the novel grain-boundary engineering approach.^14^ The tensile behavior of amorphous/amorphous nanolaminates shows the strength of the nanolaminates increases with the layer thickness decreasing, due to the interface obstruction to the shear band motion.^15^ Recently, an important breakthrough show that the heterogeneous nanolaminated metallic materials exhibit an ultrahigh hardness and excellent thermal stability, attributed to high thermal stability of the low-angle grain boundary.^6,16,17^ In order to determine how the interface structure could contribute to the composite response, large-scale molecular dynamics (MD) simulations are carried out to investigate the effect of interface structure on the deformation response.^17–21^ This result implies that atomic structure plays a key role in interface-driven plasticity. More recently, the nanotwinned Cu/HEA FeCoCrNi nanolaminate exhibit extraordinary strength and toughness.^22^ From both experiment and simulation, it was found that the interface structures at the atomic scale strongly affects deformation mechanisms.^14–21^ However, the effect of layer number on the deformation mechanism of nanotwinned Cu/HEA FeCoCrNi nanolaminates at nanoscale still remains secret.

In this work, the effect of the interface and layer number on the plastic deformation during indentation is studied in the nanotwinned Cu/HEA FeCoCrNi nanolaminate by large-scale MD simulations. To this end, we first construct a modeling of Cu/HEA FeCoCrNi nanolaminate to study the indentation response. The rest of this work can be organized in the following way. In Section 2, the model and method of MD simulation are described. In Section 3, the deformation mechanics of Cu/HEA nanolaminates as a function of layer number is characterized. Finally, in Section 4, some concluding remarks are presented.

## 2. Simulation model and method

**Fig. 1:**
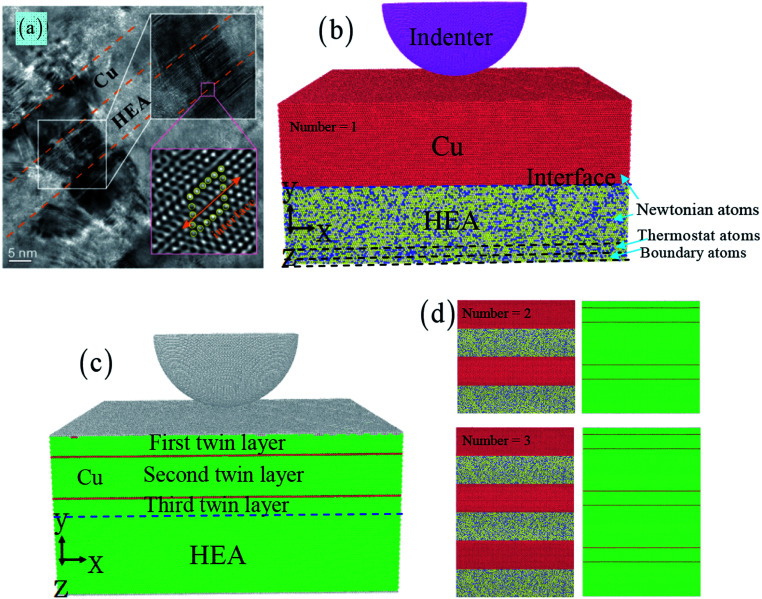
The nanotwinned Cu/HEA FeCoCrNi nanolaminates prepared by direct current magnetron sputtering deposition (a).^22^ The atomic model of nanotwinned Cu/HEA FeCoCrNi nanolaminate under the indentation test, where atoms are colored by the atom types (b). 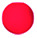 Cu, 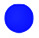 Fe, 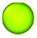 Co, 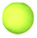 Cr, and 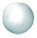 Ni. The atoms are represented by different colors based on the structure types (c). Number of 2 and 3 for the nanotwinned Cu/HEA FeCoCrNi nanolaminate (d).

Fig. 1 shows the indentation simulation of nanotwinned Cu/HEA FeCoCrNi nanolaminates, which includes the nanotwinned Cu layer, the crystalline HEA layer and the spherical diamond indenter (see Fig. 1). Based on the experiment,^22^ the dimension of the sample is 41.1 × 10 × 36.9 nm^3^ in the nanotwinned Cu layer and 41.1 × 10 × 36.9 nm^3^ in the HEA FeCoCrNi layer. The lattice parameters of the Cu layer and HEA FeCoCrNi layer are 3.69 Å and 3.59 Å, respectively, and the lattice mismatch for Cu/HEA is 2.8%. The experiment shows that most segments of a Cu/HEA interface are coherent without misfit dislocations.^22^ Our current simulation also suggests that the interface between the Cu and HEA is coherent, as shown in Fig. 1(c and d). The radius of the spherical diamond indenter is 10 nm. In addition, the effect of the nanotwinned Cu/HEA FeCoCrNi layer number on the mechanical properties and deformation behaviour would be studied. The numbers of the composite structures are chosen as 1, 2, and 3, and the corresponding atom numbers are 2 612 736, 5 225 472, and 7 838 208 (see Fig. 1(d)). The number “1” stands for the composite structure, which includes a nanotwinned Cu layer and a HEA FeCoCrNi layer (see Fig. 1(b and c)). A crystallographic orientation of first/third layer in nanotwinned Cu is [1̄10] along the *x*-axis, [111] along the *y*-axis, and [1̄1̄2] along the *z*-axis, and that of nanotwinned Cu second layer is [11̄0] along the *x*-axis, [111] along the *y*-axis, and [1̄1̄2] along the *z*-axis. The crystallographic orientation of the crystalline HEA layer is set as [1̄10] along the *x*-axis, [111] along the *y*-axis, and [1̄1̄2] along the *z*-axis. The atom of the crystalline FeCoCrNi HEA is randomly distributed with Fe, Co, Cr, and Ni atoms. The HEA layer is composed of three kinds of atoms: the boundary layer atoms with a thickness of 1.0 nm at the bottom of HEA layer are kept fixed, the thermostat layer atoms with a thickness of 1.0 nm of HEA layer adjacent to the boundary atoms are kept at a constant temperature of 300 K by the velocity scaling method, and Newtonian layer atoms meet the classic Newton's second law (Fig. 1(b)). The periodic boundary conditions are applied at *x*, and *z* directions, and the free surface is used to the *y* direction.

For the nanoindentation process, the three different atomic interactions are used: (1) the Fe–Co–Cr–Ni–Cu interaction in the Cu/HEA nanolaminate substrate is described by the embedded atom method (EAM) potential,^17–20,23–27^ which is widely used in studying the solidification, microstructure evolution, and plastic deformation driven by the loading.^25–28^ Using the EAM potential, some works have studied the lattice distortion, strain, and stress state in FCC, body centered cubic (BCC), and hexagonal close-packed (HCP) HEAs.^29–32^ Recently, the plastic response of an equi-atomic CoCrFeMnNi HEA under nanoindentation is studied by MD simulation.^33^ (2) The C–C interaction in the tool is ignored because of a rigid indenter.^34–36^ (3) The Cu (Fe, Co, Cr, Ni)–C interaction in the indenter and the Cu/HEA substrate is set by the Morse potential.^37–40^

The initial temperature of MD simulation in the nanotwinned Cu/HEA FeCoCrNi nanolaminates is 300 K. Before the indentation, the sample is first subjected to the energy minimization using the conjugate gradient method, and then all relaxed to obtain an equilibrium state. For the indentation, the constant indenter speed of 10 m s^−1^ is applied along a reverse direction of the *y*-direction. A time step of MD simulation is 1 fs. The large-scale atomic/molecular massively-parallel simulator (LAMMPS) is used for all MD simulations.^41^ The microstructural evolution is presented *via* the Ovito software.^42^ The common neighbor analysis (CNA) is used to identify the microstructure after the indentation, where red atoms represent the stacking fault, green atoms mean the FCC structure, and the white atoms are the dislocation core and other structure.

## 3. Results and discussion

**Fig. 2:**
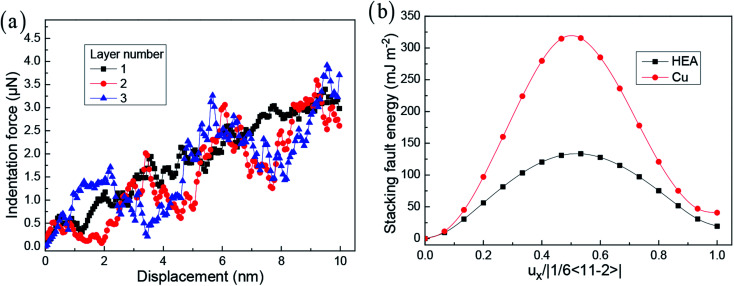
The loading force *vs.* displacement at different layer numbers (a). Generalized stacking fault energies as a function of normalized Burgers vector in HEA and Cu (b).

In previous work, the layer number strongly affects the mechanical properties and deformation behaviour during the indentation process. Fig. 2 presents the curve of loading force and indenter displacement. The hardness of materials is important for the evaluation of their mechanical properties. According to the previous work,^43,44^ the hardness *H* is calculated by *H* = *F*_max_/*A*_c_, where *F*_max_ is the maximum indentation load, and *A*_c_ is the projected area of the indenter. Here, the hardnesses of the simulated samples from their corresponding indentation curves at elastic deformation stage are 16.4, 16.8, and 19.3 GPa, respectively. As the indentation displacement increases, the indentation force dynamically increases. In addition, when the layer number increases, the softening stage and hardening stage are obviously observed. For different layer numbers, this trend presents a vastly different behaviour, owing to the interface effect to control the mechanism of the dislocation emission and the formation mechanism of the dislocation network. For the crystalline HEA, the solution strengthening effect mainly attributes to the severe-lattice-distortion enhanced resistance to a dislocation motion, and the variation of elastic modulus due to the random element distribution has a significant effect on mechanical properties.^45^ In the CuTa/Cu multilayer thin film, the solution strengthening and microstructure variation are considered to be responsible for this profound strengthening effect.^46^

**Fig. 3:**
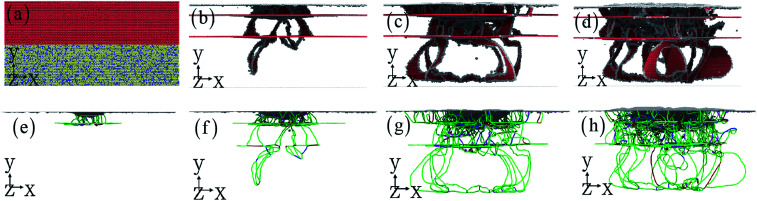
The dislocation interacting with twinning boundary at different indentation displacements. The nanotwinned Cu/HEA FeCoCrNi nanolaminate at indentation depth of 2 nm (a and e), 4 nm (b and f), 8 nm (c and g), and 10 nm (d and h). The evolution of microstructure (b–d). The dislocation structure (e–h). As indicated by the line colors, dislocations, including perfect dislocations (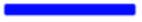 blue line), Shockley partials (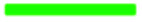 green line), Hirth (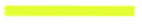 light-yellow), and stair-rod (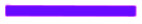 pink line) dislocations, have nonstandard Burgers vectors.

**Fig. 4:**
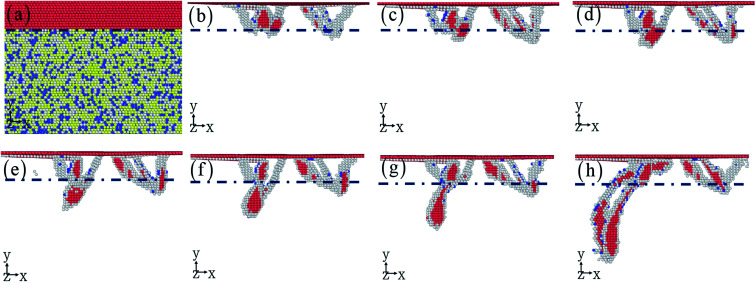
The zoomed-in views of dislocation transmission through coherent interface between HEA and Cu with the increase of indentation depth. The dark blue dotted line indicates the coherent interface.

For obtaining the robust mechanical properties, the microstructure with the increase of indentation displacement should be investigated in detail. In addition, to reveal why the violent fluctuation of indentation force occurs, the relationship between microstructure and indentation force should be presented. Fig. 3 shows the homogeneous dislocation nucleation and motion within Cu and HEA layers. The dislocations in nanotwinned layer are nucleated and emitted, which are more limited to the first twinning layer > second twinning layer > HEA layer.^22^ It is observed that Shockley partial dislocation loops are emitted from the twinning boundary into HEA layer (Fig. 3). Here, the detailed process for dislocation transmission through coherent interface is clearly observed in Fig. 4. A full dislocation is dissociated into leading and trailing partial dislocations in Cu layer, and then the leading partial dislocation is interacting with the interface. Firstly, a leading partial dislocation is stopped at the interface, and a trailing partial dislocation propagates on the same plane with the increase of indentation depth. After the leading and trailing partial dislocations propagate through the interface, the stacking faults between them widen in HEA (Fig. 3(d)), due to that the HEA has low stacking fault energy.^28,29^ The dislocation line is suddenly stretched and the width of stacking fault is increased (Fig. 3(f–h)), which occur at the coherent interface between nanotwinned Cu and HEA FeCoCrNi. Some previous simulation and theoretical work has studied the grain boundary stability on the mechanical properties of materials introduced solute elements and precipitates.^17,47,48^ The stacking fault strengthening is pronounced due to the obvious difference of stacking fault energy between Cu and HEA (see Fig. 2(b)),^29,49–51^ where the stacking fault energies of HEA and Cu are 19.4 and 40.8 mJ m^−2^. This trend is rarely observed in the previous work in traditional alloys and metals.^18–21^

**Fig. 5:**
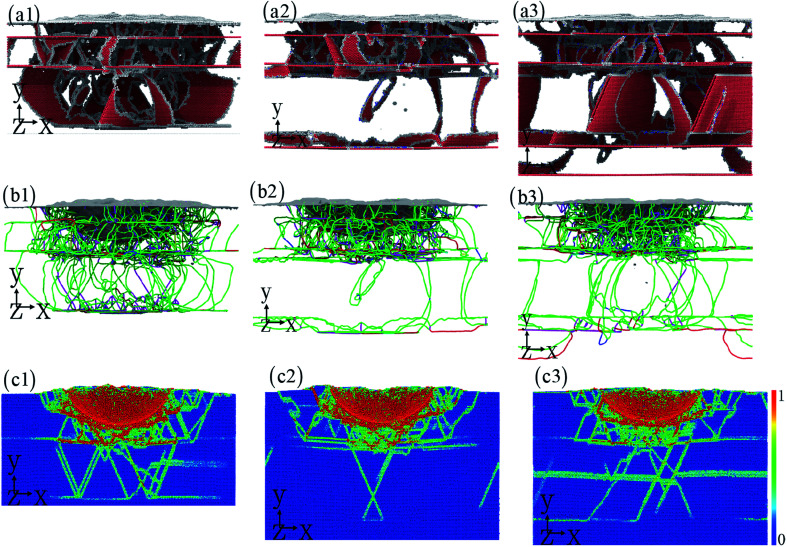
The effect of layer number: 1 (a1–c1), 2 (a2–c2), and 3 (a3–c3). The dislocation interacting with twinning boundary (a1–a3), the dislocation structure (b1–b3), and the strain distribution (c1–c3).

To understand the effect of layer number on the dislocation evolution,^52,53^ the interaction between dislocation and twinning boundary (interface) is presented in Fig. 5(a1–a3). With the increase layer number, the dislocation is mainly nucleated from twinning boundary compared to the Cu–HEA interface. A large number of dislocations take place in the nanotwinned Cu layer, resulting in the dislocation hardening of nanotwinned Cu/HEA FeCoCrNi nanolaminate. The dislocation can slip freely in HEA layer, causing the good plasticity of nanotwinned Cu/HEA FeCoCrNi nanolaminate. In addition, the dislocation substructure is also shown in Fig. 5(b1–b3). After the indentation induced deformation, the nanotwinned Cu/HEA FeCoCrNi nanolaminates for different layer numbers generate a mass of Shockley partial dislocations, which gradually decrease along the indentation direction due to the strong strain gradient effect (Fig. 5(c1–c3)).^54,55^ The strain distribution at the cross section is shown in Fig. 5(c1–c3), where the red atoms stand for the high shear strain, and the blue atoms mean the low or free shear strain. The high local strain occurs at the twinning boundary and interface as well as stacking fault, to cause the nucleation of nanocrack with the increasing indentation depth. This trend is observed experimentally in the crystalline/crystalline nanolaminates, which eventually break by the propagation of interface crack.^56,57^

## 4. Conclusions

In this work, we used atomic scale MD simulations to study the response of nanotwinned Cu/HEA FeCoCrNi nanolaminate to indentation. The results provide deep understanding in the plastic deformation mechanisms and are expected to guide the design of nanotwinned Cu/HEA FeCoCrNi nanolaminate materials with high strength and good plasticity. The dislocations are nucleated and emitted, which are more limited to the first twinning layer > second twinning layer > HEA layer. The stacking fault strengthening is pronounced due to the obvious difference of stacking fault energy between Cu and HEA, which can be rarely observed in the previous work in traditional alloys and metals. After the indentation induced deformation, the nanotwinned Cu/HEA FeCoCrNi nanolaminates for different layer numbers generate a mass of Shockley partial dislocations to cause the good plasticity, attributed to the strong strain gradient effect. Based on the above findings, this work can help to understand the role of interface, twinning boundary as well as layer number, and thus achieves for enhancing the strength and ductility in nanolaminated metals *via* tuning the interface characteristics and layer number.

## Conflicts of interest

There are no conflicts to declare.

## Supplementary Material
